# A practical guide to virtual debriefings: communities of inquiry perspective

**DOI:** 10.1186/s41077-020-00141-1

**Published:** 2020-08-12

**Authors:** Adam Cheng, Michaela Kolbe, Vincent Grant, Susan Eller, Roberta Hales, Benjamin Symon, Sharon Griswold, Walter Eppich

**Affiliations:** 1 KidSIM-ASPIRE Research Program, Alberta Children’s Hospital, Departments of Pediatrics and Emergency Medicine, Cumming School of Medicine, University of Calgary, 28 Oki Drive NW, Calgary, Canada; 2 Simulation Center, UniversitatsSpital Zurich, Ramistrasse 100, 8091 Zurich, Switzerland; 3 Center for Immersive And Simulation-based Learning, Stanford University, Stanford, USA; 4 Center for Simulation, Advanced Education and Innovation, The Children’s Hospital of Philadelphia, Philadelphia, USA; 5 Simulation Training Optimising Resuscitation for Kids (STORK), Queensland Children’s Hospital, School of Clinical Medicine, University of Queensland, Brisbane, Australia; 6 Department of Emergency Medicine, Penn State Health Milton S. Hershey Medical Center, 500 University Drive, M.C. H043, P.O. Box 850, Hershey, PA USA; 7 Departments of Pediatrics and Medical Education, Northwestern University Feinberg School of Medicine, Ann & Robert H. Lurie Children’s Hospital of Chicago, Chicago, USA

**Keywords:** Debriefing, Communities of inquiry, Virtual, Online, Remote, Learning

## Abstract

Many simulation programs have recently shifted towards providing remote simulations with virtual debriefings. Virtual debriefings involve educators facilitating conversations through web-based videoconferencing platforms. Facilitating debriefings through a computer interface introduces a unique set of challenges. Educators require practical guidance to support meaningful virtual learning in the transition from in-person to virtual debriefings. The communities of inquiry conceptual framework offer a useful structure to organize practical guidance for conducting virtual debriefings. The communities of inquiry framework describe the three key elements—social presence, teaching presence, and cognitive presence—all of which contribute to the overall learning experience. In this paper, we (1) define the CoI framework and describe its three core elements, (2) highlight how virtual debriefings align with CoI, (3) anticipate barriers to effective virtual debriefings, and (4) share practical strategies to overcome these hurdles.

The coronavirus pandemic altered the educational landscape due to social distancing policies implemented to slow the spread of disease [1–4]. As a result of this physical separation from learners, healthcare simulation programs shifted to remote simulations with virtual debriefings using web-based videoconferencing platforms [5, 6]. These necessary pedagogical adaptations have highlighted the power and potential of virtual education. However, facilitating debriefings through computer interfaces poses unique challenges since many aspects of in-person debriefing translate poorly to virtual debriefings. Educators require guidance and innovative solutions to ensure the effectiveness of simulation-based educational interventions.

In a virtual simulation, educators must facilitate the pre-brief, orchestrate clinical scenarios, and perform debriefings for learners in a remote fashion [6–10]. Virtual learning occurs synchronously and collaboratively enabling learners to interact with each other and educators in real-time, typically during lectures or group discussions [11]. We view “virtual debriefings” as a type of virtual learning, where educators use web-based video conferencing platforms to facilitate reflection in post-event discussions [7, 12–15]. The rapid shift to virtual debriefings forced simulation educators to adapt their current debriefing approaches to online platforms overnight. They require practical guidelines to support meaningful virtual learning in the transition from in-person to virtual debriefings.

Conceptual frameworks codify ways of thinking about a problem or issue, help represent complexity, and illuminate key aspects [16]. Conceptual frameworks explain, either in graphic or written form, “the main things to be studied—the key factors, concepts, or variables—and the presumed relationships among them” [17]. The communities of inquiry (CoI) framework include three core elements (cognitive, social, and teaching presence) and offers a useful structure to organize practical guidance for conducting virtual debriefings. In this paper, we aim to (1) define the CoI framework and describe its three core elements, (2) highlight how virtual debriefings align with CoI, (3) anticipate barriers to effective virtual debriefings, and (4) share practical strategies to overcome these hurdles.

## Communities of inquiry for virtual learning

A strong sense of community contributes to effective virtual learning environments [18, 19]. Originally developed for asynchronous, text-based online learning, the CoI framework has since been studied and applied extensively, with literature also supporting its applicability for synchronous online learning [20–23]. The CoI conceptual framework describes the three core interrelated elements—teacher presence, cognitive presence, and social presence—required to create successful virtual learning environments [19, 24]. Our preference is to use the term “educator’” rather than “teacher,” since in debriefings, educators often play more of a facilitative role [25]. These three elements conceptualize how online learning spaces are jointly created by the manner in which educators plan and facilitate their session, how learners think and solve problems together, and the ways in which all parties connect socially within online contexts.

*Educator presence* relates to how educators design and implement educational activities, facilitate discourse to build understanding, and provide instruction to clarify misconceptions and consolidate learning. Educators must appropriately structure and deliver content, define topics and guide discussion, share personal meaning, seek consensus, and summarize key learning points [24, 26]. All of these contribute to successful online learning experiences. How learners think together represents their *cognitive presence*, defined as the extent to which learners critically reflect and construct meaning through reflective discourse. Indicators of cognitive presence include learners who identify problems, exchange and connect ideas, brainstorm solutions, and apply new concepts to existing practice [24, 26]. Lastly, social, and emotional connections represent *social presence,* which refers to how learners “project their personal characteristics,” [24] both socially and emotionally, specifically within virtual environments. For example, a normally extroverted and gregarious person may project as shy or cautious in online environments given the different social cues. Social presence involves creating a climate in which learners communicate openly and share emotions generated by their learning experiences. Successful collaboration and shared contribution to mutual goals lead to group cohesion, which builds a sense of belonging and commitment that enables social presence [24].

These three elements are closely linked and inter-related; no element in isolation sufficiently optimizes the learning experience. For example, a skilled educator (i.e., educator presence) impacts not only the curriculum, but how learners engage and connect with each other (i.e., social presence). A warm and welcoming group culture (i.e., social presence) promotes critical thinking and reflection (i.e., cognitive presence), while thoughtful discourse can promote authentic social engagement and a culture of personal improvement [19, 24].

## Virtual debriefings as communities of inquiry

**Fig. 1:**
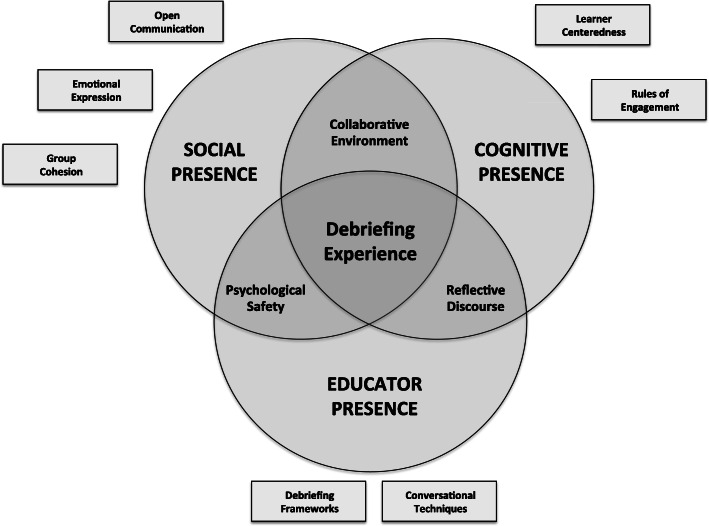
Virtual debriefing as a community of inquiry. Figure legend: Revised and adapted with permission from Garrison et al., Critical inquiry in a text-based environment: computer conferencing in higher education [24]. This Venn diagram depicts the inter-relatedness of social, cognitive, and educator presence to the overall virtual debriefing experience. Boxes represent key components within one corresponding element, while overlapping areas of the Venn diagram represent components that play an important role in two CoI elements

The CoI framework offers a useful lens for virtual debriefings since the three core elements seem to align with the current conceptualization of post-simulation debriefing. Social presence conveys the importance of interpersonal connections amongst learners, educator presence emphasizes the key role of the educator in structuring discussion, and cognitive presence highlights how reflective discourse is central to learning during debriefing. In the following sections, we will justify how the CoI framework aligns with a virtual debriefing by describing: (a) how each element and its core components are enacted during virtual debriefings, (b) barriers to successfully establishing each element in virtual learning environments, and (c) strategies to overcome these challenges. While educators can often rely on their in-person debriefing skills, some challenges will require new or seldom-used strategies to promote virtual learning. Fig. 1 is a Venn diagram representing the CoI framework adapted for virtual debriefing, depicting the inter-relatedness of the three core elements and the respective components that contribute to effective debriefing practice.

## Social presence in virtual debriefings

When learners and educators feel socially present and “real” during their online interactions [27], this comprises social presence. To socially be present, individuals should be able to project their personal characteristics and identity in online environments and contribute collaboratively to debriefing [19]. Social presence supports a high degree of interdependency as learners reflect, analyze, and synthesize learning together. Three key components contribute to the social presence, namely open communication, emotional expression, and group cohesion [21], all of which are enabled by psychological safety.

Psychological safety is the perception amongst learners that they feel safe enough to take interpersonal risks without repercussions [28, 29]. Learners must feel comfortable and safe enough to engage in open communication and contribute actively to discussions. Similarly, learners’ level of comfort supports emotional expression, which denotes sharing, understanding, and appreciating emotions in online environments [30]. Insufficient psychological safety hinders open communication and emotional expression. Educators must keep in mind that synchronous virtual learning environments likely moderate learners’ perception of psychological safety. On the one hand, virtual learning environments can promote feelings of safety because learners participate from the comfort of their own homes, lessening cues that invoke hierarchy [13, 31]. On the other hand, some learners may compensate for the limitations of virtual environments by optimizing their self-presentation, which distracts from relevant participation due to higher self-awareness [32]. Privacy concerns may also impact open communication and emotional expression. Whereas some learners participate from home, others join from public spaces with the inherent fear of being overhead, which may limit contributions to the discussion or sharing of emotions. Finally, group cohesion captures the sense of collaboration and group identity. This enables learners to air opposing views while maintaining trust, feeling acknowledged, and supported by others [30].

In summary, the social presence contributes to a shared social identity, arising from a sense of belonging and interpersonal bonds amongst leaners [33]. A psychologically safe learning environment facilitates these social connections that promote the authentic reflective discourse vital to effective virtual debriefings.

### Barriers to social presence in virtual debriefings

Multiple barriers to open communication and expressing emotion exist. Importantly, educators should not take social interactions for granted just because technology makes them possible [34]; psychological safety remains a vital ingredient. In online environments, implicit contributions to psychological safety are limited [28]. For example, educators have no physical debriefing room to thoughtfully arrange with respect to seating order, appearance, lighting, or privacy. Further, computer interfaces interfere with non-verbal cues such as body language, facial expressions, and eye contact. Whereas eye contact during in-person debriefing conveys empathy or provides validation [35], learners and educators in online environments may be unable to identify the directionality of gaze, thus muting the power of eye contact. Consequently, if learners get upset or a difficult debriefing situation evolves, educators may not react effectively, inadvertently threatening psychological safety. Also, if learners see uninvited visitors enter and leave the screen of other learners, they may perceive a lack of privacy that prevents them from sharing thoughts or emotions freely. Furthermore, managing interruptions shifts solely from educators to a shared responsibility amongst all debriefing participants.

Potential barriers to group cohesion abound. Constraints in online communication limit educators’ and learners’ use of matched body language to achieve interpersonal synchrony, which would typically increase in-person group cohesion [36]. Additionally, learners and educators usually acknowledge what has been said and understood via brief utterances such as “yeah, uh huh” [37]; however, technical constraints make this almost impossible in a virtual group setting like debriefing. The resulting frustration may reduce group cohesion and diminish engagement. Therefore, educators must substitute implicit acknowledgment with explicit verbal explanations, which requires additional cognitive effort and time on the part of educators to formulate, and learners to process. In fact, video-conferencing groups must spend more time than face-to-face groups clarifying issues and managing discussion, which demonstrates the additional effort required to achieve group cohesion in virtual environments [38].

### Strategies to enhance social presence in virtual debriefings

Building and maintaining psychological safety facilitates open communication and emotional expression in virtual environments and demands attention to explicit strategies. However, implicit strategies to build psychological safety still apply. These consist of being present (e.g., arriving early, being available for questions), kind (e.g., smiling and nodding), welcoming (i.e., using casual communication to encourage learners), and remaining attentive and interested (e.g., having eyes on the screen) [39]. Essential explicit strategies to promote psychological safety include (Table 1):

- *Conduct a briefing*. During the briefing, explicitly explain debriefing goals and process and invite learners to engage in the discussion [28]. Familiarize learners with the online learning environment and ground rules about confidentiality, privacy, and minimizing interruptions.
- *Explicitly use verbal appreciation, validation, and normalization*. These strategies help learners feel invited, acknowledged, and safe throughout the debriefing [28, 35]. See Grant et al. for details on applying these strategies during debriefing [35]. Encourage input by directing questions at certain learners. Explicitly address learners by name to help coordinate the debriefing process and convey personal regard for individual learners [40].
- *Role model fallibility and share personal experiences*. Openly sharing past failures and lessons learned from those failures helps to flatten hierarchy and promote psychological safety [28, 41].
- *Co-facilitate with another educator*. Co-facilitators may assist with cross-monitoring, facilitating recognition, and management of frustrated, angry, or upset learners [42].

**Table 1:** Strategies to enhance social presence in virtual debriefings

	Strategy	Descriptor	Benefits
**Pre-debriefing**	Conduct a briefing	Explain goals and process of debriefing, establish expectations, discuss ground rules (e.g., confidentiality, privacy)	Establishes expectations and familiarizes learners to online learning environment
**During debriefing**	Use of verbal appreciation, validation, and normalization	Acknowledge learner contributions, recognize behaviors as appropriate, relate feelings, or attitudes to a societal norm	Helps learners feel invited, acknowledged, and safe during the debriefing; promotes open communication and emotional expression.
	Role model fallibility and share past experiences	Openly sharing past failures and lessons learned	Flattens hierarchy and promotes psychological safety
	Co-facilitate	Involving a second educator to support the virtual debriefing	Facilitates cross-monitoring and divides up educator workload
	Apply implicit strategies	Be warm, kind, attentive, and welcoming	Builds psychological safety and promotes open communication and emotional expression
	Promote inclusivity	Express appreciation and use inclusive language to refer to the group	Builds group cohesion and promotes open communication
	Use explicit communication	Address participants by name, paraphrase and recap key comments	Builds group cohesion and promotes open communication

Specific strategies to deepen group cohesion in virtual debriefings:

- *Express appreciation and promote inclusivity*. Promote participants’ sense of shared social identity and their sense of belonging by expressing appreciation (e.g., “Thank you for joining this group effort ...”), also by using inclusive pronouns to refer to the group (e.g., “Let *us* talk about...” rather than “*I* want to talk to you about…”) [28, 43].
- *Utilize explicit communication strategies* [37]. This includes explicitly addressing participants by name, referring explicitly to others’ messages, paraphrasing, and asking questions [44]. Ensure that all learners have their preferred name displayed on screen to facilitate its use.

## Educator presence in virtual debriefings

Multiple factors promote educator presence, such as designing and directing instruction as well as building understanding through reflective discourse. However, our focus here is on the debriefing component of well-designed simulation-based learning experiences. To this end, we now explore how educators build understanding through reflective discourse. While virtual and in-person debriefing environments differ, basic tenets of effective debriefing still apply, such as using a structured framework for debriefing and various conversational techniques [45]. Existing debriefing frameworks have been effectively used in studies of remote simulation and virtual debriefing [9, 12–14]. Such debriefing frameworks support educator presence by structuring the discussion, defining topics, and promoting a summary of key learning points. In line with in-person debriefings, participants in virtual debriefings also recognize the value of addressing emotions, reflecting-on-action, engaging collectively, hearing differing opinions, developing shared understanding, and identifying solutions and lessons learned that can be carried over to clinical practice [13]. These processes occur during the “reactions,” “analysis,” and/or “summary/application” phases of various debriefing frameworks, demonstrating that these frameworks also apply to virtual debriefing environments [45–51]. Using a familiar debriefing framework for both in-person and virtual debriefings reinforces shared expectations of the debriefing experience for educators and learners alike.

Various techniques comprise the debriefing toolbox, such as learner self-assessment [46], directive feedback [52], advocacy inquiry [47, 53], guided team self-correction [48], and circular questions [54]. These techniques engage participants during both virtual and in-person debriefings, and educators use them intentionally based on learning context, participant goals, and their own skillset [46, 55]. Since in-person and virtual debriefings share overarching goals, simulation educators should use the skills they have, which enhances their educator presence, and thus, their ability to debrief effectively in virtual environments. We encourage educators to remain mindful of how potential barriers may affect the overall quality of discussion and adapt their approach accordingly.

### Barriers to educator presence in virtual debriefings

Educators must master key features of the teleconferencing platform in order to minimize or avoid technical issues. Technical challenges may lead to delays and disruptions that impact the quality of debriefing conversations [56, 57]. Poor video transmission impedes everyone’s ability to read facial expressions and poor audio quality influences how participants respond to comments and questions. Further, the inability to screen-share limits real-time display of key resources. Educators unfamiliar with the gallery (or grid) view feature cannot see all participants at one time, which may limit discussion. Finally, the chatbox feature for private or group messages provides an alternate mode of text-based communication that skilled educators can use strategically to enhance facilitation and/or learning [58].

Educators must also manage their attention and limit distractions. Mental workload refers to the information processing capacity required to complete a task or satisfy performance expectations [59]. When an educator’s mental workload exceeds cognitive capacity, debriefing quality and learning outcomes suffer [60]. Technical difficulties represent unnecessary work that requires mental processing but does not contribute directly to participant learning. Brower described a faculty development program designed to train educators to facilitate discourse in the online space, in which educators reported feeling “overwhelmed trying to deliver (content)… within unfamiliar technological platforms” [61]. As a consequence, technical issues hamper educator presence and distract from the relevant tasks of facilitating discussion, closing performance gaps, and summarizing learning points.

### Strategies to enhance educator presence in virtual debriefings

A number of strategies promote educator presence, some technical and some related to debriefing technique. Technical strategies include (Table 2):

- *Optimize educator visibility on video*. Sit in a well-lit room with a light source preferably from the front rather than behind, with a neutral-colored, plain background. Place the camera at an eye level by placing books under a laptop if needed and position the head centrally on the computer screen. Face the camera and look into the camera frequently to ensure perceptions of eye contact. These steps ensure that participants can read the educator’s facial expressions.
- *Test sound quality ahead of time*. Choose a quiet location and use wired headphones if needed to ensure that participants hear you clearly.
- *Use the gallery/grid view function*. This function provides a view of all participants and educators in one screen. Seeing everyone at once allows educators to pick up on non-verbal cues by seeing and scanning the “room.”
- *Use the chatbox feature (available on some platforms)*. The chatbox enables private communication between educators. While this strategy may add mental workload for educators, the added benefit of open communication and coordination amongst educators may improve the overall quality of facilitation. Another option is to use a separate messaging application not within the teleconferencing platform to avoid inadvertent comments meant for educators that appear to all participants.
- *Conduct an educator rehearsal or training session*. Educators should be trained ahead of time to troubleshoot technical issues, develop shared expectations, and distribute workload [12, 13, 57]. Doing this may mitigate the impact of technical challenges.

**Table 2:** Strategies to enhance educator presence in virtual debriefings

	Strategy	Descriptor	Benefits
**Pre-debriefing**	Conduct an educator rehearsal or training session	Session to provide educators’ opportunity to developed shared expectations and discuss how to manage technical issues	Circumvents or mitigates technical issues; provides an environment for reflective discourse
	Ensure educator is visible and check the sound quality	Educators should be clearly visible, in center of the screen, directly facing camera, and in a quiet, well-lit room, with clear sound	Optimizes educator’s ability to communicate through verbal and non-verbal channels
**During debriefing**	Co-facilitate with an educator	Involving a second educator to support the virtual debriefing	Facilitates cross-monitoring and divides up the workload
	Use gallery/grid view function	Provides a full view of all learners in a grid-like display	Most (if not all participants) can be seen on the screen all at once, allowing non-verbal cues to be detected by “scanning” the screen
	Use the chatbox features	Enables text-based communication between educators (and/or learners)	Facilitates open (and private) communication between educators (to share thoughts or coordinate tasks)
	Apply existing debriefing frameworks and conversational techniques	Frameworks help structure discussion, while conversational techniques facilitate analysis and consolidation of learning	Enhances reflective discourse while minimizing educator mental workload
	Use debriefing tools	Scripts or cognitive aids used to support the application of debriefing framework or conversational technique	Enhances reflective discourse while minimizing educator mental workload

Strategies related to debriefing technique include:

- *Co-facilitate with a second educator*. Co-facilitations allow for workload distribution [42]. For example, one educator can manage the technical aspects of the virtual environment such as playing video, monitoring the chatbox, or muting participants while the other focuses on facilitating discussion.
- *Apply existing debriefing frameworks and conversational techniques*. These frameworks and strategies help structure discussion, promote reflection, identify solutions, and summarize key learning points.
- *Use debriefing tools (as necessary)*. Debriefing tools can support the implementation of debriefing frameworks or conversational techniques and may reduce educator mental workload by providing scripted phrases to trigger reflection and discussion [60, 62].

## Cognitive presence in virtual debriefings

Cognitive presence refers to the learners’ ability to construct and confirm meaning through reflective discourse [19, 24]. As in face-to-face debriefing, simulation scenarios serve as the springboard for virtual exploration, reflection, and learning. Skillful educators who model openness and encourage diverse points of view while facilitating structured discussion build learners’ cognitive presence [21]. Virtual debriefings also benefit from educators who adeptly manage the debriefing process to prompt reflection while balancing learner vs. instructor-centeredness. In particular, learner-centered facilitation approaches to enhance the cognitive presence through a reflective discourse that encourages learners to identify and explore problems, exchange knowledge and debate ambiguities, connect ideas and identify solutions, and apply new concepts and solutions. Thus, learners take collaborative responsibility for their own learning by selecting content and guiding the pace [21] and flow of discussion [25].

To optimize cognitive presence, learners should feel comfortable collaborating virtually and using strategies to engage with other learners and educators. Educators must explicitly clarify in advance the rules of engagement for virtual debriefings, such as turn taking during the conversation, using technical features such as hand-raising to indicate a desire to speak, ensuring attentiveness, and minimizing interruptions while others are speaking. In particular, we recommend that all participants keep themselves muted unless they are actively contributing to avoid unintended distractions. As a sign of cognitive presence, learners may search the internet for information, which may assist with information gathering and augment discussion if it does not limit engagement.

### Barriers to cognitive presence in virtual debriefings

The cognitive presence relies on educators who are familiar with debriefing frameworks and conversational techniques. Beyond these basics, we see two other key barriers to learner cognitive presence during virtual debriefings: (1) lack of familiarity with rules of engagement or technical aspects of online, collaborative environments and (2) excess learner cognitive load. Synchronous online learning is not intuitive to all, as many learners describe difficulty engaging with others during online discussion [20, 58, 63]. Some learners do not know how to engage in this new social context, with technology-induced barriers to building a collaborative environment. A poor internet connection may result in delays or poor audio and video quality; multiple individuals speaking at once may result in awkward pauses, and interruptions within one individuals’ home environment such as the appearance of a child may disrupt the flow of discussion. Some learners may perform concurrent tasks during the debriefing such as searching for content or checking email, which detracts from cognitive presence [64].

Cognitive load theory describes how learners must “attend to, manipulate, and understand the information in a conceptualized area of the brain known as working memory” [60] in order to learn something new. Extraneous load are tasks that require mental processing but do not directly contribute to enhancing learning. Since working memory is limited, new information, ideas, or concepts may be lost if extraneous load detracts from learners’ working memory capacity [64]. Learners participating in synchronous online learning described technological difficulties and distractions as an extraneous load that negatively impacted learning [64]. Some reported “requiring guidance from their instructor as the platform was not self-explanatory”. This evidence highlights the need for educators to adopt strategies to minimize the impact of an extraneous load.

### Strategies to enhance cognitive presence in virtual debriefings

We suggest several strategies to enhance cognitive presence (Table 3):

- *Apply learner-centered debriefing strategies*. Identify the learner’s agenda, work with learners to prioritize content for discussion, promote learner self-assessment, and encourage learners to close performance gaps [25].
- *Orient learners to the collaborative environment*. Provide learners with an orientation to the features of the online environment, including how to mute themselves, how to activate gallery/grid view, how to use the chatbox (if permitted), and how to share their screen [64]. Share expectations in advance, such an appropriate audio and speaker setup (headphones ideal) and a quiet space to minimize interruptions and ensure confidentiality.
- *Communicate rules of engagement*. Establish rules of engagement collaboratively with learners, including the use of the raise hand feature to indicate a desire to speak, muting microphone when not actively contributing, emphasizing the importance of privacy and confidentiality (i.e., no screen recordings), establishing an expectation of turn taking when speaking to promote maximal engagement by all learners, and determining how/if the chatbox will be used as an alternate means of sharing comments or questions [64].
- *Reduce learner cognitive load*. Engage both audio and visual channels during interactions^65^ through the use of an online “whiteboard” or screen-sharing to make notes visible to all learners during the debriefing (e.g., visually populating two columns in a plus-delta exercise) during the discussion. To reduce extraneous load, learners should be encouraged to refrain from activities that are not directly related to the discussion. This can be accomplished by closing their email application, silencing their phone, and shutting off instant messaging programs.
- *Using online breakout rooms*. Smaller group breakout sessions may be an option to enhance cognitive engagement and active discussion [64].

**Table 3:** Strategies to enhance cognitive presence in virtual debriefings

	Strategy	Descriptor	Benefits
**Pre-debriefing**	Conduct a briefing	Orient the learner to the collaborative environment—how to mute, how to activate gallery view, how to use the chatbox	Establishes expectations and familiarizes learners to the online learning environment
	Communicate rules of engagement	Sharing expectations for how to communicate, including the use of raise hand feature, speaking up, emphasizing privacy and confidentiality, waiting for others to finish speaking before you speak, muting computer when not speaking, close distractions (email, phone) in the work area	Learners clearly understand how to communicate in the virtual environment; contributes to establishing a collaborative learning environment
**During debriefing**	Apply learner-centered debriefing strategies	Learner-centered strategies encourage learners to identify problems, connect ideas, identify solutions, and apply new concepts	Promotes engagement of learners and reflective discourse; contributes to establishing a collaborative learning environment
	Reduce learner cognitive load	Excess cognitive load can impede learners’ ability to retain new ideas or concepts	Promotes engagement of learners and reflective discourse
	Use online breakout rooms	Separate virtual breakout rooms permit smaller group discussion	Promotes engagement of learners and reflective discourse; contributes to establishing a collaborative learning environment

## Conclusion

The community of inquiry framework aligns well with the unique demands of virtual debriefing. Three inter-related core elements of social, educator, and cognitive presence offer a valuable organizing principle for practical guidance. While educators can rely on existing debriefing frameworks and conversational techniques, they must place increased emphasis on explicit strategies to build and maintain psychological safety. Further, educators must manage learner cognitive load to maximize mental capacity for the relevant reflective discourse that promotes learning after simulations.

## Data Availability

Not applicable.

## Abbreviations

CoI: Communities of Inquiry
